# Rapid on-site monitoring of *Legionella pneumophila* in cooling tower water using a portable microfluidic system

**DOI:** 10.1038/s41598-017-03293-9

**Published:** 2017-06-08

**Authors:** Nobuyasu Yamaguchi, Yusuke Tokunaga, Satoko Goto, Yudai Fujii, Fumiya Banno, Akiko Edagawa

**Affiliations:** 1 Osaka Institute of Public Health, 1-3-69 Nakamichi, Higashinari, Osaka, 537-0025 Japan; 2 Graduate School of Pharmaceutical Sciences, Osaka University, 1-6 Yamada-oka, Suita, Osaka, 565-0871 Japan

## Abstract

Legionnaires’ disease, predominantly caused by the bacterium *Legionella pneumophila*, has increased in prevalence worldwide. The most common mode of transmission of *Legionella* is inhalation of contaminated aerosols, such as those generated by cooling towers. Simple, rapid and accurate methods to enumerate *L. pneumophila* are required to prevent the spread of this organism. Here, we applied a microfluidic device for on-chip fluorescent staining and semi-automated counting of *L. pneumophila* in cooling tower water. We also constructed a portable system for rapid on-site monitoring and used it to enumerate target bacterial cells rapidly flowing in the microchannel. A fluorescently-labelled polyclonal antibody was used for the selective detection of *L. pneumophila* serogroup 1 in the samples. The counts of *L. pneumophila* in cooling tower water obtained using the system and fluorescence microscopy were similar. The detection limit of the system was 10^4^ cells/ml, but lower numbers of *L. pneumophila* cells (10^1^ to 10^3^ cells/ml) could be detected following concentration of 0.5–3 L of the water sample by filtration. Our technique is rapid to perform (1.5 h), semi-automated (on-chip staining and counting), and portable for on-site measurement, and it may therefore be effective in the initial screening of *Legionella* contamination in freshwater.

## Introduction

Legionnaires’ disease in pneumonic form and Pontiac fever were first recognised in 1976. Since then, the number of cases of Legionnaires’ disease has increased worldwide, particularly in the United States (https://www.cdc.gov/legionella/surv-reporting.html) and Europe (http://ecdc.europa.eu/en/data-tools/Pages/home.aspx). In Japan, the number of cases doubled between 2010 and 2015 (http://www.nih.go.jp/niid/ja/data.html). However, the number of patients is thought to be under-reported^1^ because the early symptoms of Legionnaires’ disease are similar to those of a common cold and quite number of patients are treated by empirical chemotherapy without diagnostic confirmation as Legionnaires’ disease^2^.

Legionnaires’ disease is often referred to as ‘traveller’s disease’ with the rise in domestic and international travel. Surveillance for this travel-associated disease has increased in the United States and Europe. Being a volcanic country, Japan has many spa resorts with hot springs. Many Japanese and foreign travellers visit and enjoy a culture of bathing in these hot springs. However, not a few of these spa facilities use filtration and circulation systems for the bathing water, and these artificial systems often cause an increase in *Legionella* cells following the increase in free-living amoebas in the systems^3^. In July 2002, a large outbreak of Legionnaires’ disease occurred in a bathhouse with spa facilities in Miyazaki Prefecture, Japan^4, 5^. About 300 patients suffered pneumonia and/or symptoms of fever and a cough, with seven fatalities. This outbreak also caused serious economic damage to the resort area.

It is worth noting that 75–80% of patients with Legionnaires’ disease are over 50 years of age (http://www.who.int/mediacentre/factsheets/fs285/en/), and therefore it is assumed that the number of patients will increase in accordance with aging of the population in many developed countries.

There have been 60 species of *Legionella* reported to date, which have comprised 70 distinct serogroups^6^; however, *Legionella pneumophila* is the predominant species (>90% of cases) isolated from patients with Legionnaires’ disease^7^. Among the 16 serogroups of *L. pneumophila*, the primary serogroup associated with outbreaks is serogroup 1 (http://www.who.int/water_sanitation_health/emerging/legionella.pdf). The most common form of transmission of *L. pneumophila* is inhalation of contaminated aerosols, which may occur from cooling towers, public fountains, water distribution systems and baths that circulate water^8^. Therefore, the microbial management of these systems is vital in preventing outbreaks linked to poorly maintained artificial water systems. Culture-dependent methods, such as ISO 11731, are usually employed to detect *Legionella* cells in samples; however, these methods require 10–14 days to obtain results and cannot detect viable but non-culturable (VBNC) *Legionella* cells^9^. In addition, the presence of other bacteria interferes with *Legionella* growth on media and can result in underestimation of the presence of *Legionella* cells^10, 11^. Therefore, rapid quantitative methods, which do not rely on culturing, are required to enumerate *Legionella* cells, and gene-targeting techniques such as PCR are now widely applied. However, these techniques require extraction of DNA or RNA, which is rather difficult to perform in the field. Instead, *L. pneumophila* cells can be rapidly and accurately detected by culture-independent techniques at a single cell level, such as flow cytometry following immunomagnetic separation and fluorescent staining^12, 13^, and on-site monitoring of *Legionella* cells would be effective for the microbial management of artificial water systems, the most common sites of *Legionella* outbreaks.

A microfluidic device is a small device containing microchannels that has been developed during decades of progress in microfabrication technologies. Microfluidic device-based analyses are rapid and are performed on a smaller scale, thereby consuming less sample and reagents than conventional approaches^14^; thus these devices have great potential in environmental microbiology^15–18^. Microfluidic devices can reduce the biohazard risk because cells are analysed in a closed system and the devices are immediately sterilised after use, making these devices suitable for application in public and environmental health microbiology settings. However, most microfluidic devices have been developed to separate target microbes^19, 20^ or analyse their characteristics rather than to determine the total number of target microbes^21^.

In this study, we applied our microfluidic device^22^ to enumerate *L. pneumophila* cells in cooling tower water. This microfluidic device was originally designed for on-chip fluorescent staining and semi-automated counting of target microbial cells. We also constructed a portable system for rapid on-site monitoring of *L. pneumophila* in the water samples and used it to enumerate target microbial cells rapidly flowing in the microchannel.

## Results and Discussion

*Legionella* cells in freshwater are usually detected using culture-dependent techniques such as ISO 11731; however, it can take 10–14 days to obtain results, and *Legionella* cells in a VBNC state cannot be detected. One approach to solve this problem is to develop new protocols to culture these hard-to-culture *Legionella* cells^11^. Another approach is to develop culture-independent techniques that can rapidly detect an increase in *Legionella* cells and also estimate the microbiological properties of freshwater that is potentially contaminated by *L. pneumophila* ^12, 13, 23^. We therefore investigated the possibility of combining a microfluidic device with a portable system for rapid on-site monitoring of *L. pneumophila* in cooling tower water.

### Concentration of low-abundance *L. pneumophila* cells in cooling tower water samples

The number of *L*. *pneumophila* cells in freshwater is usually not high enough for detection. In Japan, the control limit of *L. pneumophila* in cooling tower water is 100 colony-forming units (CFU)/100 ml (2) and the concentration of the cells is usually required. In addition, one of the cooling tower water samples collected in this study (Supplementary Fig. S1B) contained algal cells and detritus that clogged the flow in the microchannel of the microfluidic device (Supplementary Fig. S2). Therefore, for reliable enumeration of *Legionella* cells in cooling tower water, these inhibitors need to be removed and *Legionella* cells need to be concentrated in the sample. By incorporating the following steps: (i) pre-filtration with a 3-μm-pore-size filter, (ii) concentration of the filtrate with a 0.2-μm-pore-size polycarbonate filter, and (iii) resuspension in particle-free water, the recovery rates of *L. pneumophila* ATCC 33152 and *L. pneumophila* isolated from cooling tower water (*L. pneumophila* CT1 A-1) inoculated into cooling tower water as 10^3^ cells/ml were 82 ± 14% (n = 5) and 76 ± 24% (n = 5), respectively. Algal cells and detritus in the sample were effectively removed (Supplementary Fig. S2). However, by incorporating the following steps–(i) concentration with a 0.2-μm-pore-size polycarbonate filter, (ii) resuspension of trapped aggregates on the filter in particle-free water, and (iii) filtration with a 3-μm-pore-size filter—the recovery rate of inoculated *Legionella* cells decreased to below 40% (31 ± 14%; n = 5). This may be explained by the fact that *Legionella* cells adsorbed onto algal cells and/or detritus in the cooling tower water during concentration and were then removed together by filtration with the 3-μm-pore-size filter. We therefore proposed the following protocol for effective concentration of *Legionella* cells and removal of inhibitors: first, pre-filtration of 0.5–3 L of the sample with a 3-μm-pore-size filter; then concentration of the bacterial cells in the filtrate onto a 0.2-μm-pore-size polycarbonate filter; and finally resuspension of the bacterial cells trapped on the filter in 3 ml of particle-free water by vortexing for 1 min.

### On-chip staining and enumeration of *L. pneumophila* cells

**Figure 1:**
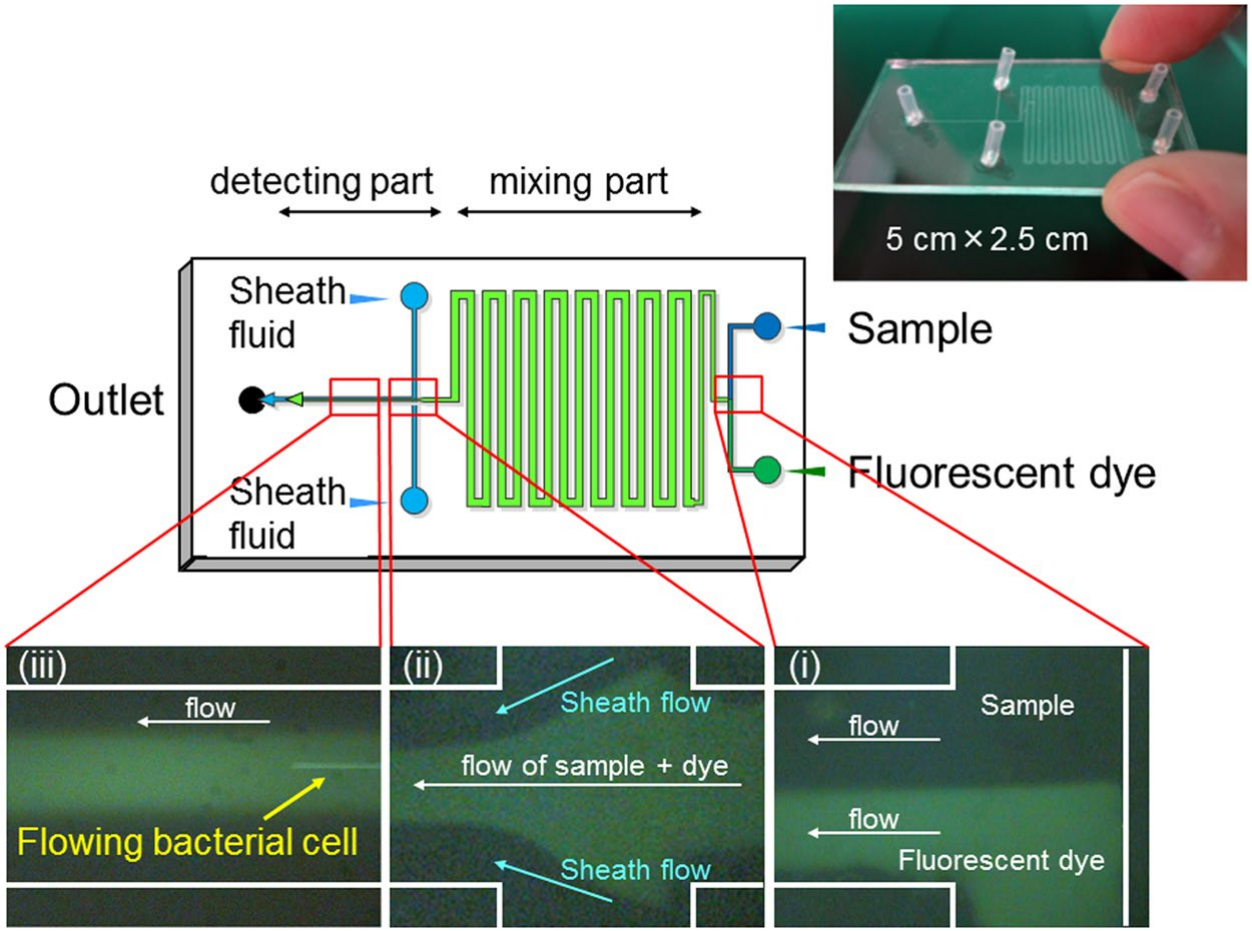
Detail of the microfluidic device for on-chip staining and counting Long and short lengths are 50 mm and 25 mm, respectively. Width of the channel is 100 μm, with the exception of the “mixing part” (500 μm). Depth of the channel is 15 μm. Sample, fluorescent dye solution and sheath fluid were injected at each inlet. The process of on-chip staining and counting of bacterial cells is shown and includes: (i) the sample and fluorescent dye solution flowing separately and becoming mixed in the “mixing part” of the microchannel, (ii) alignment of the sample flow by the sheath fluid, and (iii) the flow of bacterial cells in the “detecting part” of the microchannel.

Next, the possibility of on-chip staining of *L. pneumophila* cells with a fluorescent antibody was examined using the originally-designed microfluidic device for on-chip staining and counting (Fig. 1). It was confirmed that the sample and fluorescent antibody solution flowed separately in the microchannel at first (Fig. 1(i)); however, these solutions became mixed during flow through the “mixing part” of the microchannel (Fig. 1(ii)). Sheath fluid was effective for alignment of the flowing bacterial cells (Fig. 1(ii)), and antibody-labelled cells could be detected in the “detecting part” (Fig. 1(iii)).

**Figure 2:**
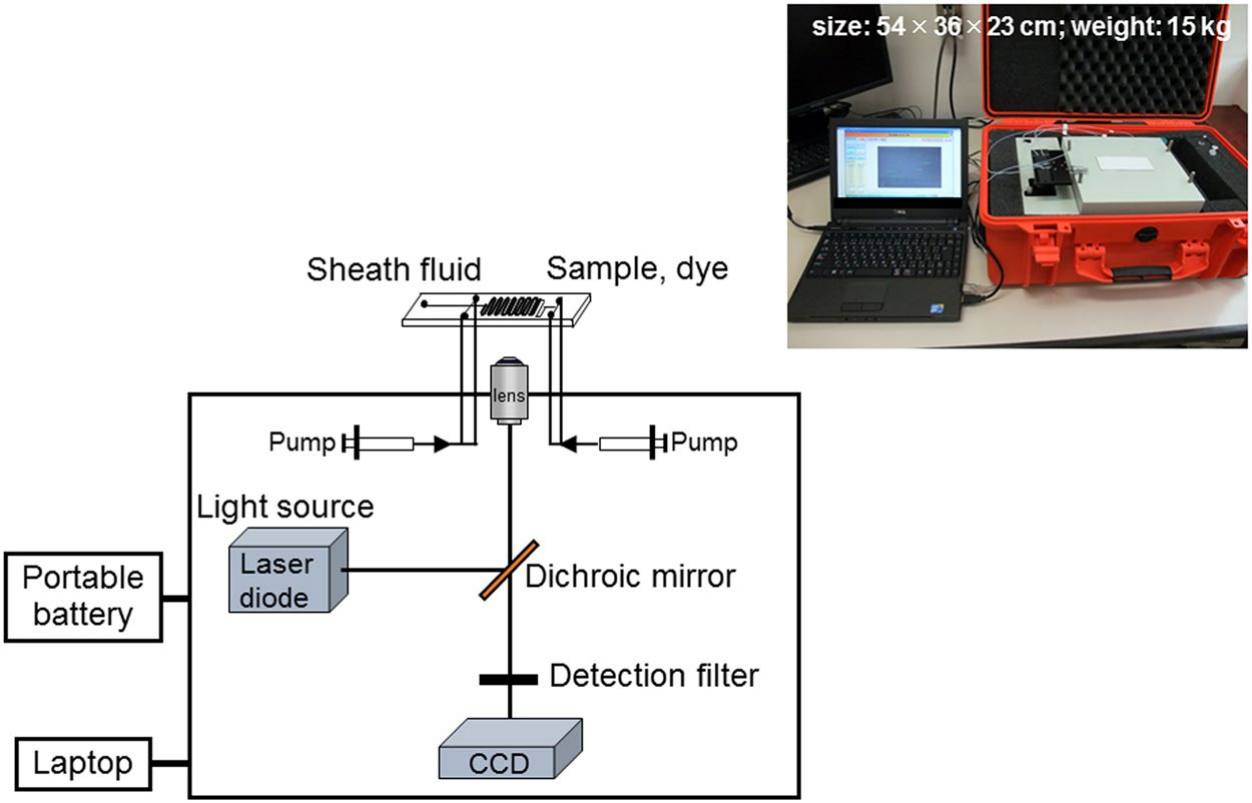
Portable microfluidic system for on-site bacterial monitoring.

For the enumeration of fluorescently-stained cells that were flowing through the microchannel of the microfluidic device, a portable system was designed and constructed (Fig. 2). This system consisted of three component parts for i) solution application (syringe pump), ii) detection (optical system with a diode laser, a filter block and a CCD camera), and iii) analysis (laptop). The system was packed into a carry case (final size: 54-cm width, 36-cm depth and 23-cm height; final weight: 15 kg). A diode laser was selected to obtain a bright blue beam of low voltage, for excitation of the fluorescently-labelled bacterial cells. This system can be set at the sampling point for rapid on-site monitoring of target bacterial cells (Supplementary Fig. S3(i)).

The concentration of fluorescent antibody for on-chip staining was optimised with 1 × 10^5^ cells/ml of *L. pneumophila* cells spiked in PBS. The microfluidic count was (9.2 ± 1.8) × 10^4^ cells/ml when 20 μg/ml of fluorescent antibody was used (n = 5), compared with (7.3 ± 2.6) × 10^4^ and (8.5 ± 2.9) × 10^4^ cells/ml when 10 and 50 μg/ml of fluorescent antibody were used, respectively. Therefore, 20 μg/ml of fluorescent antibody was selected for use in the following experiments.

**Figure 3:**
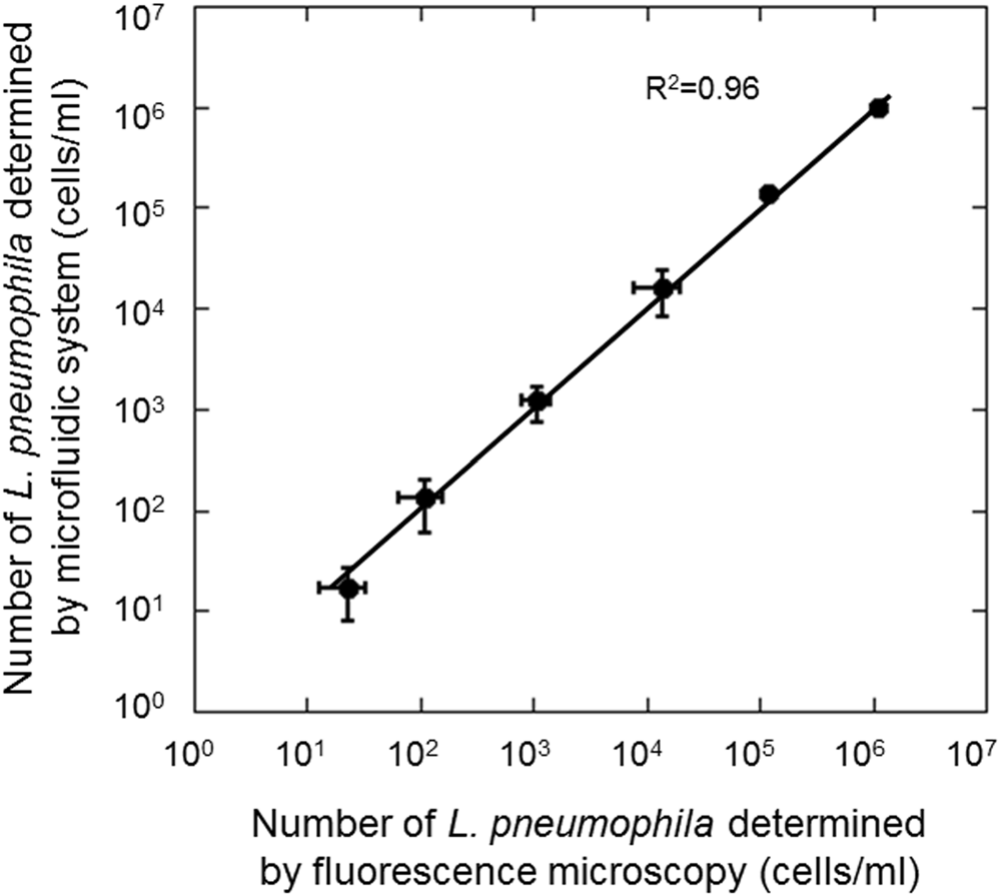
Correlation between microfluidic counts and conventional fluorescence microscopic counts of *L. pneumophila* stained with a fluorescent antibody. Cultured *L. pneumophila* cells were spiked in cooling tower water. Error bars indicate the standard deviation (n = 5). Samples with 10^1^ cells/ml of *Legionella* cells were counted following 1000-fold concentration by filtration. Samples with 10^2^ and 10^3^ cells/ml of *Legionella* were 100-fold concentrated before counting, and samples with 10^4^ to 10^6^ cells/ml of *Legionella* were counted without concentration.

Using this microfluidic device and portable system, the correlation between microfluidic counts and conventional fluorescent microscopic counts was determined for samples that contained *L. pneumophila* within the range of 10^1^ to 10^6^ cells/ml (Supplementary Table S1), and the counts of *Legionella* cells obtained by the system and by fluorescence microscopy closely correlated (Fig. 3). The detection limit of the microfluidic technique (10^4^ cells/ml) could be improved to 10^1^ cells/ml by concentration of *L. pneumophila* cells by filtration and resuspension in particle-free water with the procedure determined in this study. The maximum threshold for detection was 10^6^ cells/ml because some of these cells were not accurately counted by the portable system if too many cells (>10^6^ cells/ml) flowed rapidly in the microchannel of the device.

### Monitoring of *L. pneumophila* in cooling tower water using a microfluidic system

Following the confirmation of accuracy of the portable microfluidic system to enumerate *L. pneumophila* cells at low abundance, monitoring of *L. pneumophila* in cooling tower water was performed.

First, the accuracy of the microfluidic counts determined by the microfluidic device for on-chip staining and counting was evaluated by the desktop microfluidic system previously developed in our laboratory (Supplementary Fig. S4)^24^ because the sensitivity of this previous system was higher than the portable microfluidic system constructed in this study. Cooling tower water samples were collected at the cooling tower (A) (Supplementary Fig. S1) and *L. pneumophila* cells in the samples were concentrated by filtration and resuspended in particle-free water as previously described. The microfluidic counts were similar with the fluorescent microscopic counts (Supplementary Fig. S5) and we confirmed that the microfluidic device for on-chip staining and counting can be used for efficient counting of *L. pneumophila* cells not only in spiked samples but also in natural water samples.

**Figure 4:**
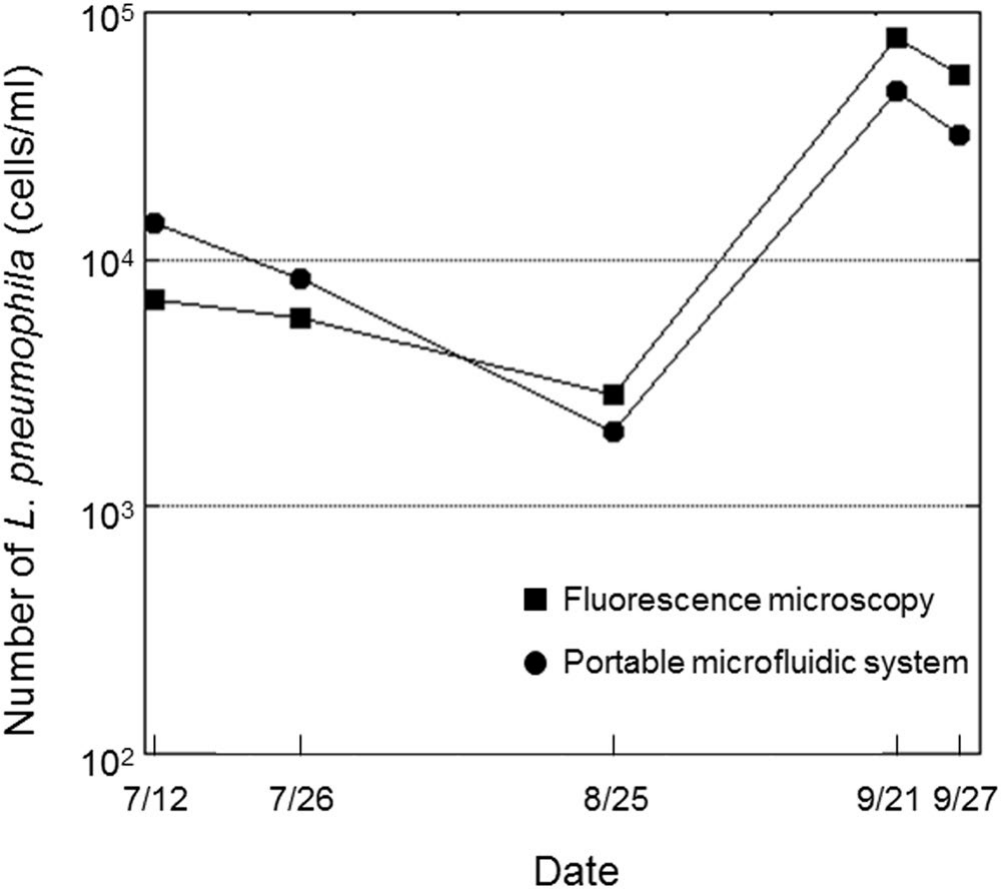
Monitoring of *L. pneumophila* in cooling tower water by fluorescence microscopy and the portable microfluidic system.

Then monitoring of *L. pneumophila* in cooling tower water was performed by the combined use of the microfluidic device for on-chip staining and counting and the portable microfluidic system (Fig. 4). Cooling tower water samples were collected at the cooling tower (B) (Supplementary Fig. S1). The existence of *Legionella* spp. in these cooling tower water samples was confirmed using a loop-mediated isothermal amplification kit. The number of culturable *L. pneumophila* was found to be 30–4800 CFU/ml during the monitoring period, which was 0.22–40.7% that of the microfluidic counts, and this lack of correlation was consistent with previous studies using culture-independent techniques^10, 12^. This is likely because fluorescent staining methods can detect *L. pneumophila* cells in the VBNC state whereas culture-dependent methods cannot^13^. It was revealed that the *L. pneumophila* cell count decreased during continuous work and disinfection (from 12 July to 25 August); however, it increased after intermittent circulation of the cooling tower water was performed and the frequency of disinfection was decreased (after 21 September). Increase and decrease of *L. pneumophila* cells could be monitored by the portable microfluidic system as well as conventional fluorescence microscopy, and the results were obtained within 1.5 h (1 h for pre-filtration of cooling tower water and concentration of *Legionella* cells and 30 min for on-chip staining and counting by the portable system). These results indicated that rapid and accurate counting of *L. pneumophila* cells in cooling tower water can be performed and appropriate microbiological control of cooling tower water can be confirmed on-site by the combined use of the microfluidic device and the portable system described in this study. Furthermore, combination of this system with an electric vehicle would expand its usefulness because it would enable easy transport of the system to a monitoring site and provide a highly energetic electric power supply outside of a laboratory (Supplementary Fig. S3(ii)).

We constructed our microfluidic device using rapid prototyping and replica-moulding techniques^24^; however, it is now possible to construct such devices using a consumer-grade 3D printer^25^, making this type of technology more readily accessible.

## Conclusion

In this study, we applied a microfluidic device and a portable system to address a problem in the field of public and environmental health microbiology. Our technique effectively enumerated total *L. pneumophila* cells without culturing and could therefore be applied for “first screening” purposes in the microbial quality control of cooling tower water and other freshwater potentially contaminated by *Legionella* cells. In this study, we used a polyclonal antibody for detection of *L. pneumophila*. One limitation of this method is that the polyclonal antibody may react with other serogroups of *L. pneumophila* and other *Legionella* species, and potentially other bacterial species. However, as an initial screening method, avoiding false-negative results is more important than the detection of false-positive cells, and using a polyclonal antibody is therefore permissible. To more specifically detect target cells, an appropriate monoclonal antibody could be selected that would attach to unique epitopes on the target bacterial surface^12^.

Our technique offers the following advantages: (i) rapid performance (1.5 h to complete); (ii) ease of use as a semi-automated system (for on-chip staining and counting); and (iii) portability for on-site measurement. A specific feature of this system that differs from gene-targeting techniques^26–28^ or electrochemical detection techniques^29^ is that it can visualise *Legionella* cells in the sample. By watching a video of the flow in the microchannel and recognising the frequency of flowing *Legionella* cells, one can directly grasp the increase or decrease in target cells even without having specialist microbiology knowledge. For example, one can recognise the increase of *L. pneumophila* cells when the frequency of flowing cells in the microchannel increases to 10 cells/min from the more regular level of 1 cell/min. This feature is important for the rapid examination of *Legionella* cells in environmental samples. In addition, fluorescent vital staining^30^ can be applied in our system to distinguish between live and dead *Legionella* cells, though this would require a colour-sensitive CCD camera with high-resolution for reliable detection. Viable *L. pneumophila* cells can be successfully distinguished from non-viable cells by combination of a nucleic acid-staining dye (SYBR Green) with propidium iodide, which can be used to detect membrane-damaged cells^13^. If a combination of 5-cyano-2,3-ditolyl tetrazolium chloride (CTC) to detect respiring cells^31^, and green fluorescently-labelled antibody to detect target cells, is applied (double staining with fluorescent antibody and CTC)^32^, active *Legionella* cells show orange fluorescence (as a result of mixing the green fluorescence from the fluorescent antibody and the red fluorescence from CTC) under blue excitation, whereas inactive *Legionella* cells show only the green fluorescence of the fluorescent antibody. Respiring bacterial cells other than *L. pneumophila* show only the red fluorescence of CTC, meaning that it is possible to distinguish active *Legionella* cells from inactive *Legionella* cells or active non-*Legionella* cells based on their fluorescence^32^.

When enumerating *L. pneumophila* cells, a greater number of inhibitors are present in cooling tower water than in other sources of freshwater, such as public fountains, bathtub water or water distribution systems, indicating that our technique may be successfully applied to these other types of environmental water samples. In fact, our technique could detect *L. pneumophila* cells in a natural river located in Kagoshima Prefecture, Japan, where discharged hot spring water and cold river water were mixed and pooled (Supplementary Fig. S6)^33^. Also, our technique could detect *L. pneumophila* cells in circulating bathtub water samples (Supplementary Fig. S7)^34^ that contained scurf and water scale (different inhibitors from cooling tower water for detection). In addition, our technique can be used to monitor various harmful bacteria by incorporating a suitable fluorescent antibody for the target bacteria. We therefore propose that our findings contribute towards technical progress in developing effective quality control systems for various sources of freshwater used for human consumption, recreation, food preparation and industry^35–37^.

## Materials and Methods

### Bacterial strains and cooling tower water

*Legionella pneumophila* ATCC 33152 and *L. pneumophila* CT1 A-1 (isolate from the cooling tower (B) in Supplementary Fig. S1) were cultured on WYOα agar media (Eiken Chemical, Tokyo, Japan). Resulting colonies were suspended in phosphate-buffered saline (PBS; 130 mM NaCl, 10 mM Na_2_HPO_4_, 10 mM NaH_2_PO_4_; pH 7.2) within the range of 10^1^ to 10^6^ cells/ml and were used in this study. The numbers of *L. pneumophila* cells suspended in PBS were determined by fluorescence microscopy as described later. To enumerate *Legionella* cells using the portable microfluidic system, samples containing 10^1^ cells/ml of *Legionella* cells were 1000-fold concentrated by filtration before counting, samples with 10^2^ and 10^3^ cells/ml of *Legionella* cells were 100-fold concentrated, and 10^4^ to 10^6^ cells/ml of *Legionella* cells were counted without concentration.

Cooling tower water samples were collected at a cooling tower located in Osaka, Japan (Supplementary Fig. S1). Cooling tower (A) was in operation throughout the year whereas cooling tower (B) was only in operation periodically. Cooling tower (B) started work on 1 July, 2016 and the cooling tower water was disinfected once a week during operation. Intermittent circulation of the cooling tower water was performed from 19 September, 2016 and the frequency of disinfection was decreased after this intermittent circulation.

Before staining of *L. pneumophila* cells in the cooling tower water with a fluorescent antibody, 0.5–3 L of water sample were filtered through a 3-μm-pore-size cellulose acetate filter (Advantec, Tokyo, Japan) to remove algal cells and detritus that may inhibit the flow of bacterial cells in the microchannel. Bacterial cells in the filtrate were then concentrated onto a 0.2-μm-pore-size polycarbonate filter (Advantec) by filtration and resuspended in 3 ml of particle-free water by vortexing for 1 min. This procedure was based on the official method used in Japan^2^ to concentrate and recover *L. pneumophila* cells in cooling tower water.

### Detection of *L. pneumophila* in cooling tower water

Culturable *L. pneumophila* cells were detected according to the procedure described in the Guideline for Prevention of Legionnaires’ Disease 3^rd^ Version^2^. This procedure was based on ISO 11731. Bacterial cells in 500 ml of cooling water sample were filtered through a 0.2-μm-pore-size polycarbonate filter (Advantec) and resuspended in 5 ml of sterilised deionised water by vortexing for 1 min. This concentrate was treated by acidification under pH 2.0 for 5 min at room temperature (approximately 25 °C) or by heating at 50 °C for 30 min, and then 0.2 or 0.1 ml of the sample was smeared onto WYOα agar medium. After incubation at 37 °C for 7 days, colonies that had formed on the WYOα agar medium were transferred to B-CYEα agar medium (Eiken Chemical) and sheep blood agar (Eiken Chemical) and then incubated for 3 days at 37 °C for confirmation of *L. pneumophila* cells by an agglutination test kit (*Legionella* Latex Test; Denka Seiken, Tokyo, Japan), which is used as an official test in Japan^2^. The detection limit of this culture method was 10 CFU/100 ml of the tested water sample.

Loop-mediated isothermal amplification (LAMP), which perform amplification and detection of target gene in a single step by incubating the mixture of sample, primers, DNA polymerase with strand displacement activity and substrates at a constant temperature (in the region of 65 °C), was also used for the specific detection of *Legionella* spp. in the samples based on their 16 S rRNA sequence (Loopamp *Legionella* Detection Kit *E*; Eiken Chemical; http://loopamp.eiken.co.jp/e/products/legionella_e/). Amplification of target gene was monitored by a realtime turbidimeter (LA-320C; Eiken Chemical).

### Fluorescent antibody and blocking solution

An anti-*L. pneumophila* antibody (6051; ViroStat, Portland, ME, USA) was labelled with an Alexa Flour 488 Protein Labelling Kit (Thermo Fisher Scientific, Waltham, MA, USA) according to the manufacturer’s instructions.

Blocking solution for antibody staining of *Legionella* cells was prepared with bovine serum albumin (BSA; Wako Pure Chemical Industries, Osaka, Japan); BSA was dissolved in PBS (final concentration: 12%) and filtered through a 0.2-μm-pore-size filter just before use to remove small particles.

### Fluorescence microscopy

The number of *L. pneumophila* in each sample was determined by fluorescence microscopy to obtain precise bacterial numbers to compare to the number determined by the microfluidic system. For blocking, BSA was added to the sample at 3% (w/v) and incubated for 15 min at room temperature (approximately 25 °C). Then, fluorescent antibody (final concentration: 4 μg/ml) was added and cells were stained for 15 min at room temperature in the dark. Stained cells in the sample were filtered onto a black polycarbonate membrane filter (pore size: 0.2 μm, diameter: 25 mm; Advantec). The stained cells were counted at a magnification of 1000X(objective lens: Plan Fluor 100X; Nikon, Tokyo, Japan) under blue excitation (Nikon B-2A cube; excitation filter EX 450–490, dichroic mirror DM 505, absorption filter BA 520) using an epifluorescent microscope (E-800; Nikon).

### Microfluidic device designed for “on-chip” staining and counting

Polydimethylsiloxane (PDMS)-glass hybrid microfluidic devices were constructed using rapid prototyping and replica-moulding techniques^24^. The masks for the channel patterns were printed onto transparent film. Ultra-thick photoresist film (SU-8-50; Microchem, Newton, MA, USA) was spin-coated onto a silicon wafer and baked on a hot plate at 65 °C for 2 min and 95 °C for 3 min. The pattern on the mask was photolithographically transferred to the SU-8-coated silicon wafer using a mask aligner (M-1S; Mikasa, Tokyo, Japan). After development in SU-8 developer (Microchem) for 3 min, the master was washed in isopropyl alcohol and then distilled water. The prepolymer of PDMS and the curing agent (Silpot 184; Toray Dow Corning, Tokyo, Japan) were mixed at a ratio of 10:1, stirred thoroughly, and then degassed under vacuum. The prepolymer mixture was poured onto the master and cured at 120 °C for 40 min. After being cured, the PDMS replica was peeled off from the master. Access ports were drilled into the device by a paper punch (diameter, 2 mm). The PDMS replica was attached to a cover glass using a plasma reactor (SEDE/V; Meiwa Fosis, Osaka, Japan). Each sample was mixed with BSA (final concentration 6%), and 20 μg/ml of fluorescent antibody and sheath fluid (particle-free water) were injected at each inlet (Fig. 1). The depth of the microchannel was 15 μm and the width was 100 μm, with the exception of the “mixing part” (500-μm width).

### Portable microfluidic system

Samples and staining fluid were placed in 100 μl gastight syringes (1710LT; Hamilton, Reno, NV, USA) and sheath fluid was placed in 1-ml gastight syringes (1001LT; Hamilton). These fluids were injected into the microfluidic device via Teflon tubes by the syringe pumps of the system (Fig. 2). Stained cells flowing in the microchannel were monitored through an objective lens (UPlanApo 40X; numerical aperture: 0.85; OLYMPUS, Tokyo, Japan) under blue excitation by a diode laser (wavelength: 473 nm; power: 60 mW) and recorded as a video using a CCD camera (WAT-902H_2_; Watec, Yamagata, Japan) for 10–15 min per sample. A filter block for an epifluorescent microscope was equipped to detect signals from the fluorescent antibody selectively (Olympus U-MNB2 cube consisted of a dichroic mirror 520IF and absorption filter 500). The flow rate was 0.01 μl/min for the sample and staining fluid, compared with 0.005 μl/min for the sheath fluid. Flowing cells in the movie were processed and counted using image analysis software (BADICS-FCM; Lambda Vision, Kanagawa, Japan), which can enhance positive signals and discriminate background fluorescence by binarisation. The number of *L. pneumophila* in each sample was calculated as cells/ml, as determined from the cell count and flow volume.

## Electronic supplementary material

Supplementary Figures and Table

## References

[1] Marston BJ, Lipman HB, Breiman RF (1994). Surveillance for Legionnaires’ disease. Risk factors for morbidity and mortality. Arch Intern Med.

[2] Meguro, K. Guideline for Prevention of Legionnaires’ Disease - Third Version. (Building Management Education Center, 2009).

[3] Ohno A, Kato N, Yamada K, Yamaguchi K (2003). Factors influencing survival of *Legionella pneumophila* serotype 1 in hot spring water and tap water. Appl Environ Microbiol.

[4] Okada M (2005). The largest outbreak of legionellosis in Japan associated with spa baths: epidemic curve and environmental investigation. Kansenshogaku Zasshi.

[5] Sasaki T (2008). An outbreak of Legionnaires’ disease associated with a circulating bathwater system at a public bathhouse. I: a clinical analysis. J Infect Chemother.

[6] Ishizaki N (2016). *Legionella thermalis* sp. nov., isolated from hot spring water in Tokyo, Japan. Microbiol Immunol.

[7] Yu VL (2002). Distribution of *Legionella* species and serogroups isolated by culture in patients with sporadic community-acquired legionellosis: an international collaborative survey. J Infect Dis.

[8] Edagawa A (2008). Detection of culturable and nonculturable *Legionella* species from hot water systems of public buildings in Japan. J Appl Microbiol.

[9] Steinert M, Emödy L, Amann R, Hacker J (1997). Resuscitation of viable but nonculturable *Legionella pneumophila* Philadelphia JR32 by *Acanthamoeba castellanii*. Appl Environ Microbiol.

[10] Delgado-Viscogliosi P (2005). Rapid method for enumeration of viable *Legionella pneumophila* and other *Legionella* spp. in water. Appl Environ Microbiol.

[11] Edagawa A (2015). Investigation of Legionella contamination in bath water samples by culture, amoebic co-culture, and real-time quantitative PCR methods. Int J Environ Res Public Health.

[12] Füchslin HP, Kötzsch S, Keserue HA, Egli T (2010). Rapid and quantitative detection of *Legionella pneumophila* applying immunomagnetic separation and flow cytometry. Cytometry A.

[13] Keserue HA, Baumgartner A, Felleisen R, Egli T (2012). Rapid detection of total and viable *Legionella pneumophila* in tap water by immunomagnetic separation, double fluorescent staining and flow cytometry. Microb Biotechnol.

[14] Blankenstein G, Larsen UD (1998). Modular concept of a laboratory on a chip for chemical and biochemical analysis. Biosens Bioelectron.

[15] Liu WT, Zhu L (2005). Environmental microbiology-on-a-chip and its future impacts. Trends Biotechnol.

[16] Bridle H, Miller B, Desmulliez MP (2014). Application of microfluidics in waterborne pathogen monitoring: A review. Water Res.

[17] Rusconi R, Garren M, Stocker R (2014). Microfluidics expanding the frontiers of microbial ecology. Annu Rev Biophys.

[18] Wu F, Dekker C (2016). Nanofabricated structures and microfluidic devices for bacteria: from techniques to biology. Chem Soc Rev.

[19] Lee J (2013). Synthetic ligand-coated magnetic nanoparticles for microfluidic bacterial separation from blood. Nano Lett.

[20] Lee, W. *et al*. 3D-printed microfluidic device for the detection of pathogenic bacteria using size-based separation in helical channel with trapezoid cross-section. *Sci Rep* **5**, Article number: 7717, doi:10.1038/srep07717 (2015). 10.1038/srep07717 PMC4289896 25578942

[21] Sakamoto C, Yamaguchi N, Nasu M (2005). Rapid and simple quantification of bacterial cells by using a microfluidic device. Appl Environ Microbiol.

[22] Yamaguchi N, Torii Y, Uebayashi Y, Nasu M (2011). Rapid, semiautomated quantification of bacterial cells in freshwater by using a microfluidic device for on-chip staining and counting. Appl Environ Microbiol.

[23] Baba T, Inoue N, Yamaguchi N, Nasu M (2012). Rapid enumeration of active *Legionella pneumophila* in freshwater environments by the microcolony method combined with direct fluorescent antibody staining. Microbes Environ.

[24] Sakamoto C (2007). Rapid quantification of bacterial cells in potable water using a simplified microfluidic device. J Microbiol Methods.

[25] Comina G, Suska A, Filippini D (2014). Low cost lab-on-a-chip prototyping with a consumer grade 3D printer. Lab Chip.

[26] Okuno T, Tani K, Yamaguchi N, Nasu M (2015). Expression of *gyrB* and 16S ribosomal RNA genes as indicators of growth and physiological activities of *Legionella pneumophila*. Biocont Sci.

[27] Ichijo, T., Yamaguchi, N., Tanigaki, F., Shirakawa, M. & Nasu, M. Four-year bacterial monitoring in the International Space Station – Japanese Experiment Module “Kibo” with culture-independent approach. *npj Microgravity* **2**, Article number 16007, doi:10.1038/npjmgrav.2016.7 (2016). 10.1038/npjmgrav.2016.7 PMC5515537 28725725

[28] Park, J., Ichijo, T., Nasu, M. & Yamaguchi, N. Investigation of bacterial effects of Asian dust events through comparison with seasonal variability in outdoor airborne bacterial community. *Sci Rep* **6**, Article number: 35706, doi:10.1038/srep35706 (2016). 10.1038/srep35706 PMC5071759 27761018

[29] Tokel, O. *et al*. Portable microfluidic integrated plasmonic platform for pathogen detection. *Sci Rep* **5**, Article number: 9152, doi:10.1038/srep09152 (2015). 10.1038/srep09152 PMC4371189 25801042

[30] Yamaguchi N, Nasu M (1997). Flow cytometric analysis of bacterial respiratory and enzymatic activity in the natural aquatic environment. J Appl Microbiol.

[31] Rodriguez GG, Phipps D, Ishiguro K, Ridgway HF (1992). Use of a fluorescent redox probe for direct visualization of actively respiring bacteria. Appl Environ Microbiol.

[32] Yamaguchi N, Sasada M, Yamanaka M, Nasu M (2003). Rapid detection of respiring *Escherichia coli* O157:H7 in apple juice, milk, and ground beef by flow cytometry. Cytometry.

[33] Yamaguchi, N. Real-time and on-site monitoring of harmful microbes in aquatic environments by microfluidic system. *Progress Reports of the Lake Biwa - Yodo River Water Quality Preservation Organization*. http://www.byq.or.jp/josei/h24/pdf/h24_seikahoukoku07.pdf.

[34] Yamaguchi, N. On-site monitoring of harmful bacteria in river environments by portable system. *Progress Reports of the River Foundation*. 26-1215-022. http://public-report.kasen.or.jp/261215022.pdf.

[35] Kawai M (2002). 16S ribosomal DNA-based analysis of bacterial diversity in purified water used in pharmaceutical manufacturing processes by PCR and denaturing gradient gel electrophoresis. Appl Environ Microbiol.

[36] Baba T, Yamaguchi N, Matsumoto R, Nasu M (2009). Bacterial population dynamics in a reverse-osmosis water purification system determined by fluorescent staining and PCR-denaturing gradient gel electrophoresis. Microbes Environ.

[37] Yamaguchi N, Ichijo T, Nasu M (2011). Environmental disease: environmental alteration and infectious disease. Ecol Res.

