# Supplementary material for: Rapid on-site monitoring of Legionella pneumophila in cooling tower water using a portable microfluidic system
Source: Sci Rep. 2017 Jun 8;7:3092. doi: 10.1038/s41598-017-03293-9 (PMC5465085; doi:10.1038/s41598-017-03293-9)
Supplement: Supplementary file 1 — Supplementary Figures and Table [file 41598_2017_3293_MOESM1_ESM.pdf]

# Supplementary Information

Rapid on-site monitoring of *Legionella pneumophila* in cooling tower water using a portable microfluidic system

Nobuyasu Yamaguchi[1, 2]*, Yusuke Tokunaga[1], Satoko Goto[2], Yudai Fujii[2], Fumiya Banno[2] and Akiko Edagawa[1]

[1] Osaka Prefectural Institute of Public Health, 1-3-69 Nakamichi, Higashinari, Osaka 537-0025, Japan

[2] Graduate School of Pharmaceutical Sciences, Osaka University, 1-6 Yamada-oka, Suita, Osaka 565-0871, Japan

*Corresponding should be addressed to N. Y. (nyyamaguchi@iph.pref.osaka.jp)

Supplementary Figures 1-7 and Supplementary Table S1

Cooling tower (A) Cooling tower (B)

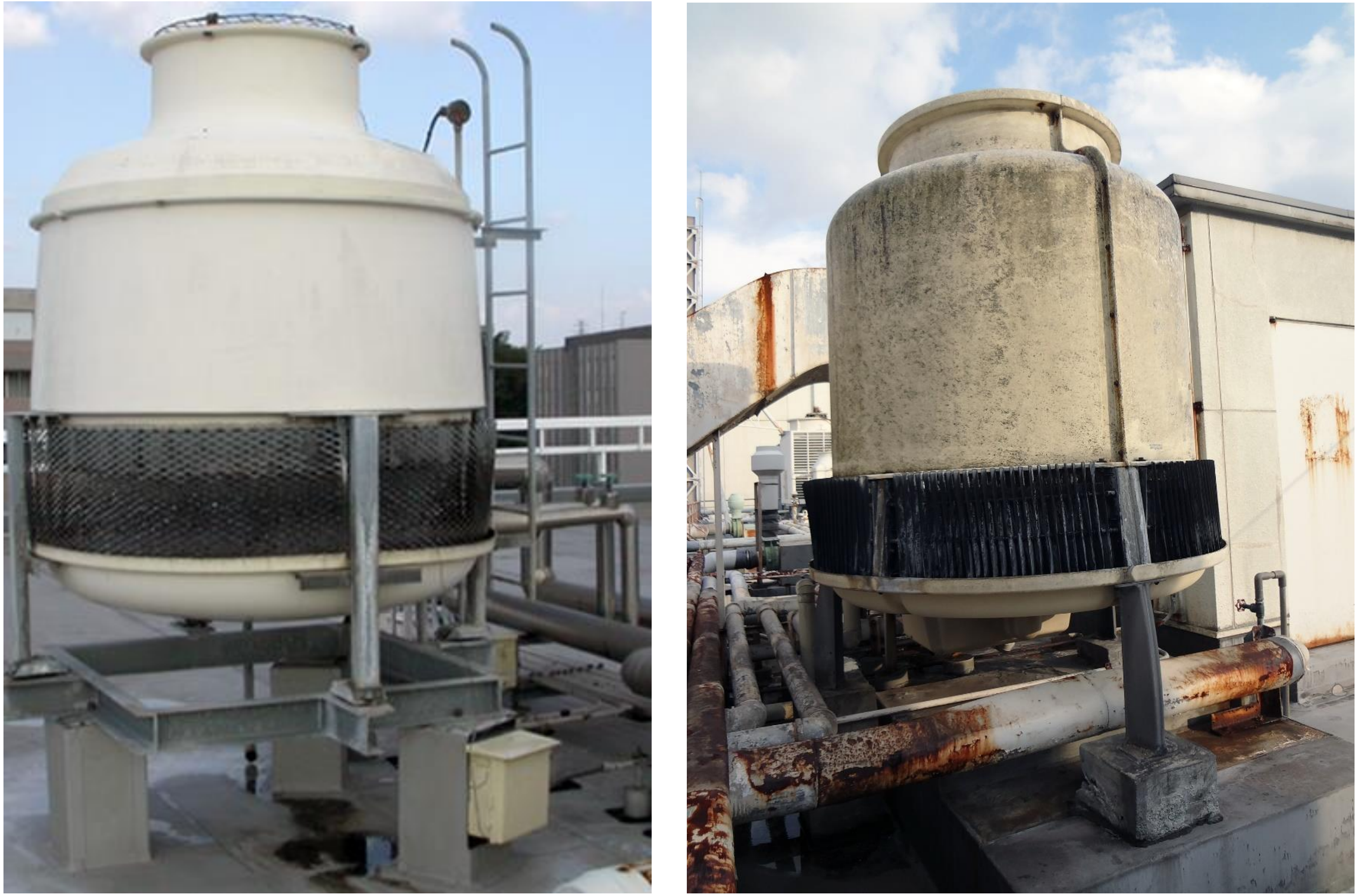

Supplementary Figure S1. Cooling towers at sampling site.

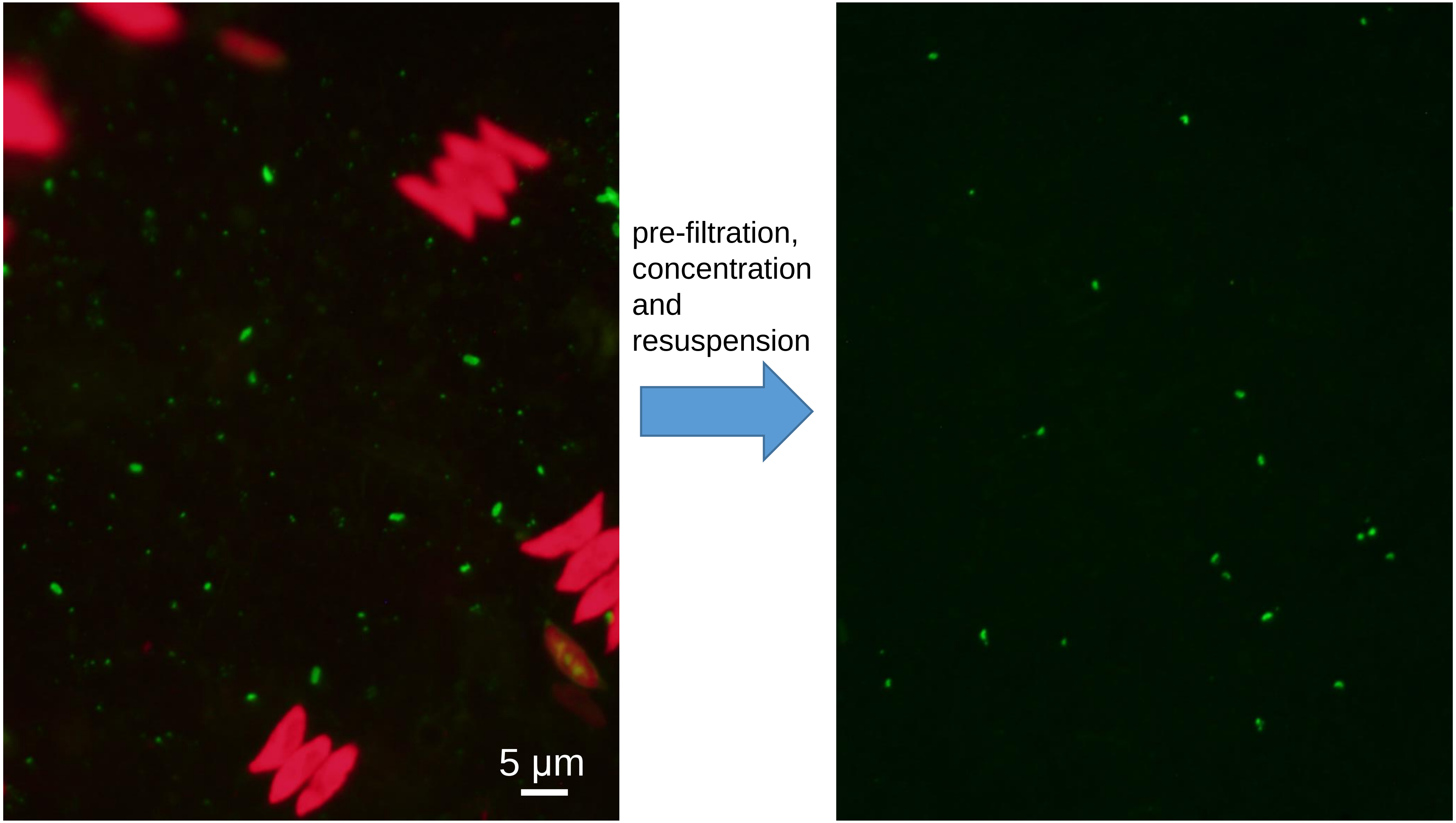


Supplementary Figure S2. Algal cells in the cooling tower water sample that fluoresce red under blue excitation and removal of algal cells by the filtration procedure determined in this study.

(i) With portable battery (ii) In electric vehicle

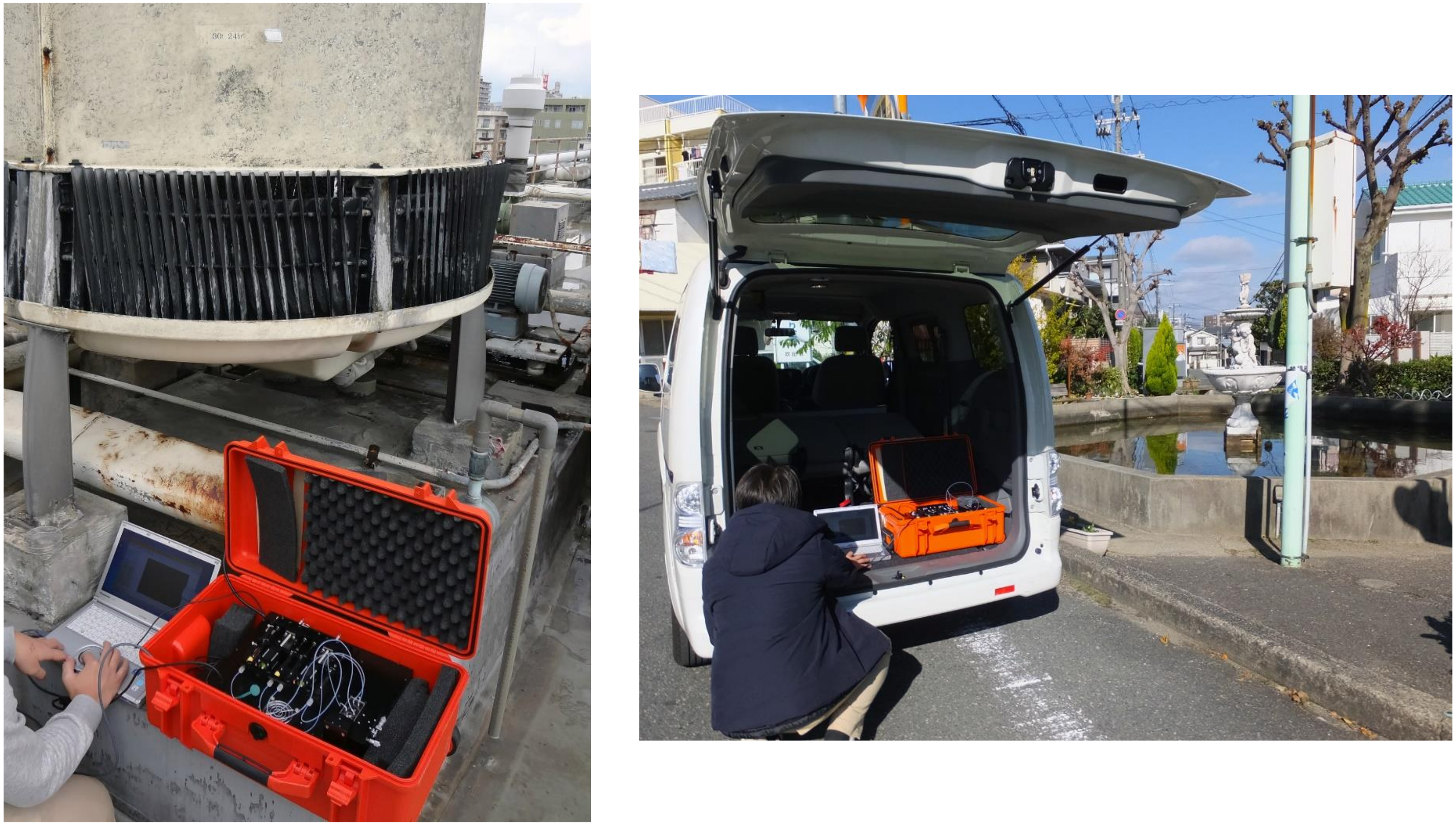

Supplementary Figure S3. On-site monitoring of *L. pneumophila* in freshwater environment using the portable microfluidic system with a portable battery (i) and in a electric vehicle (ii).

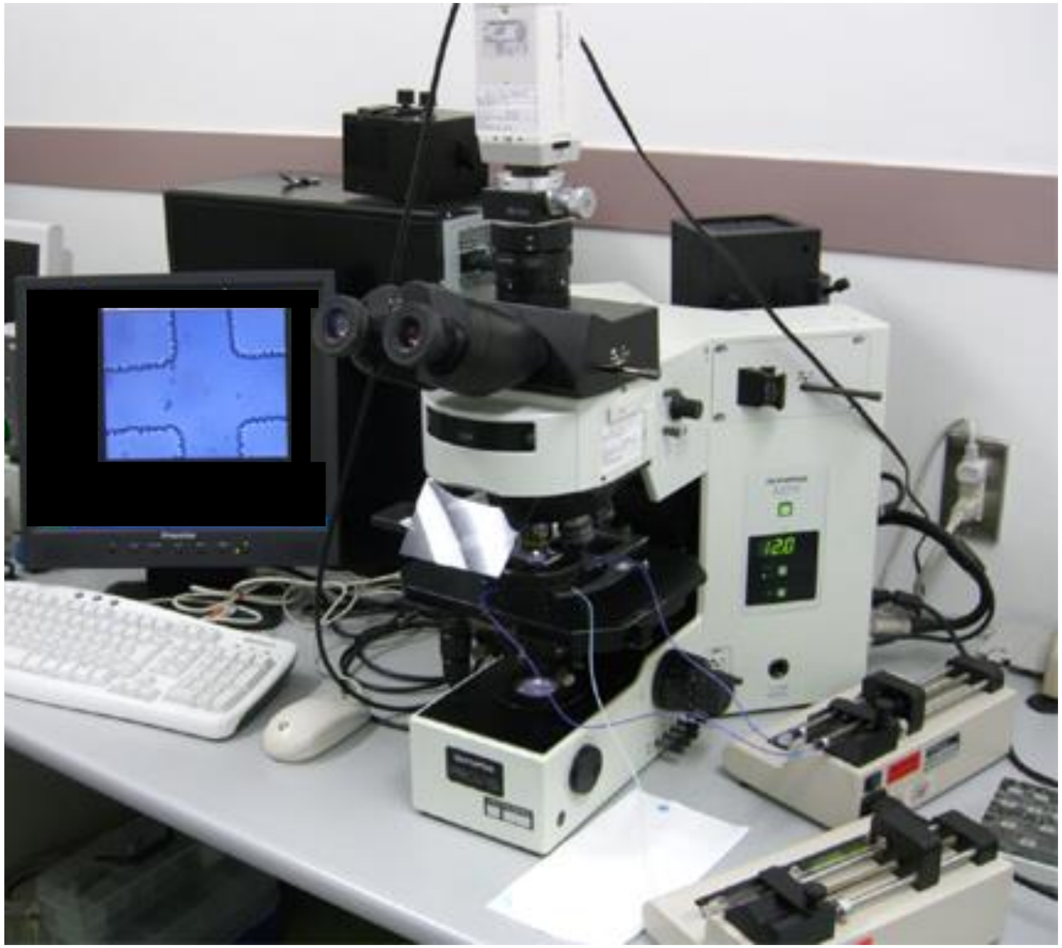

Supplementary Figure S4. Desktop microfluidic system for bacterial monitoring.

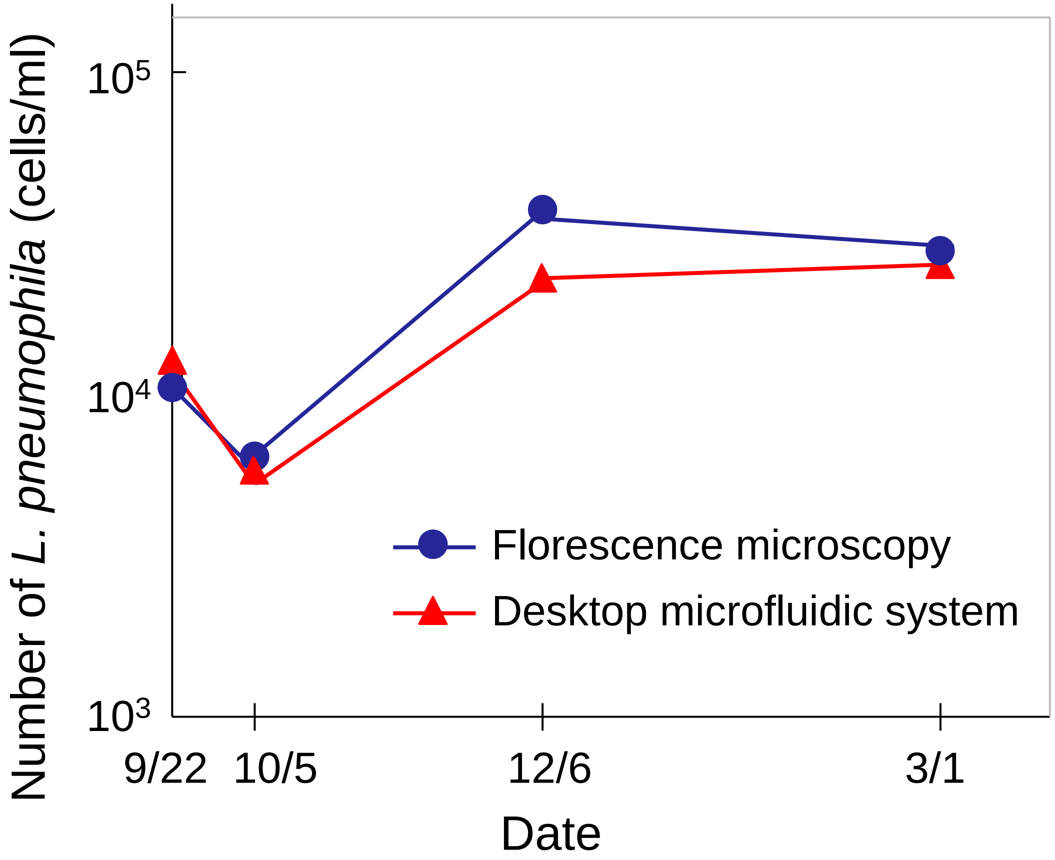


Supplementary Figure S5. Monitoring of *L. pneumophila* in cooling tower water by fluorescence microscopy and the desktop microfluidic system.

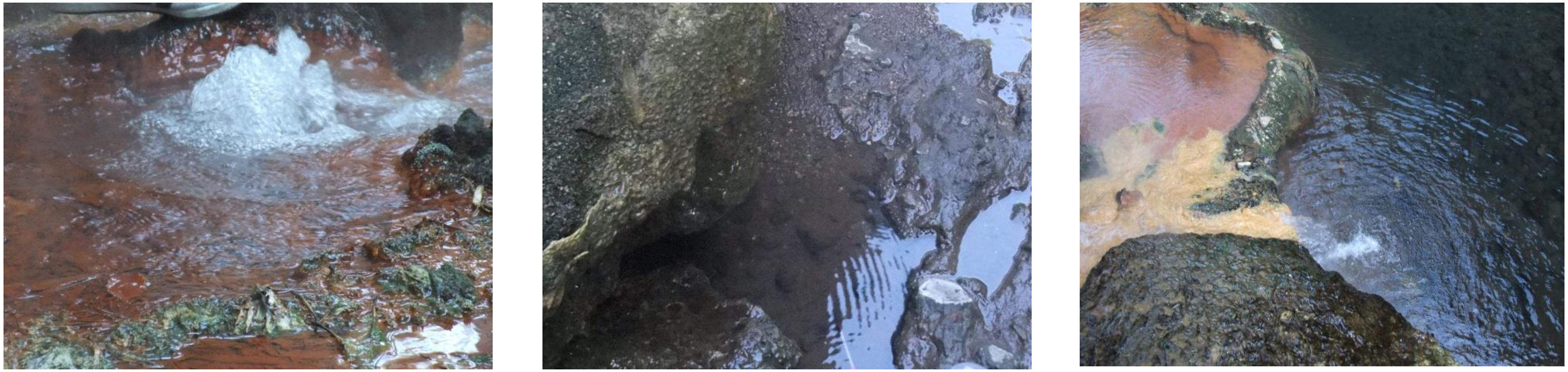

A: Hot spring

B: Pool of mixture of hot spring water and river water

C: River

| Site | Water temperature (°C) | Total bacteria[1] (cells/ml) | *L. pneumophila*[2] (cells/ml) |
|---|---|---|---|
| A | > 50 | $< 1.0 \times 10^4$ | $< 1.0 \times 10^2$ |
| B | 30 | $2.1 \times 10^5$ | $5.2 \times 10^2$ |
| C | < 15 | $4.0 \times 10^6$ | $< 1.0 \times 10^2$ |

Determined with SYBR Green staining[1] and fluorescent antibody staining[2]

Supplementary Figure S6. Monitoring of *L. pneumophila* in a natural river by the portable microfluidic system. The sampling point was located in Kagoshima Prefecture, Japan, where discharged hot spring water (A) and cold river water (C) were mixed and pooled (B).

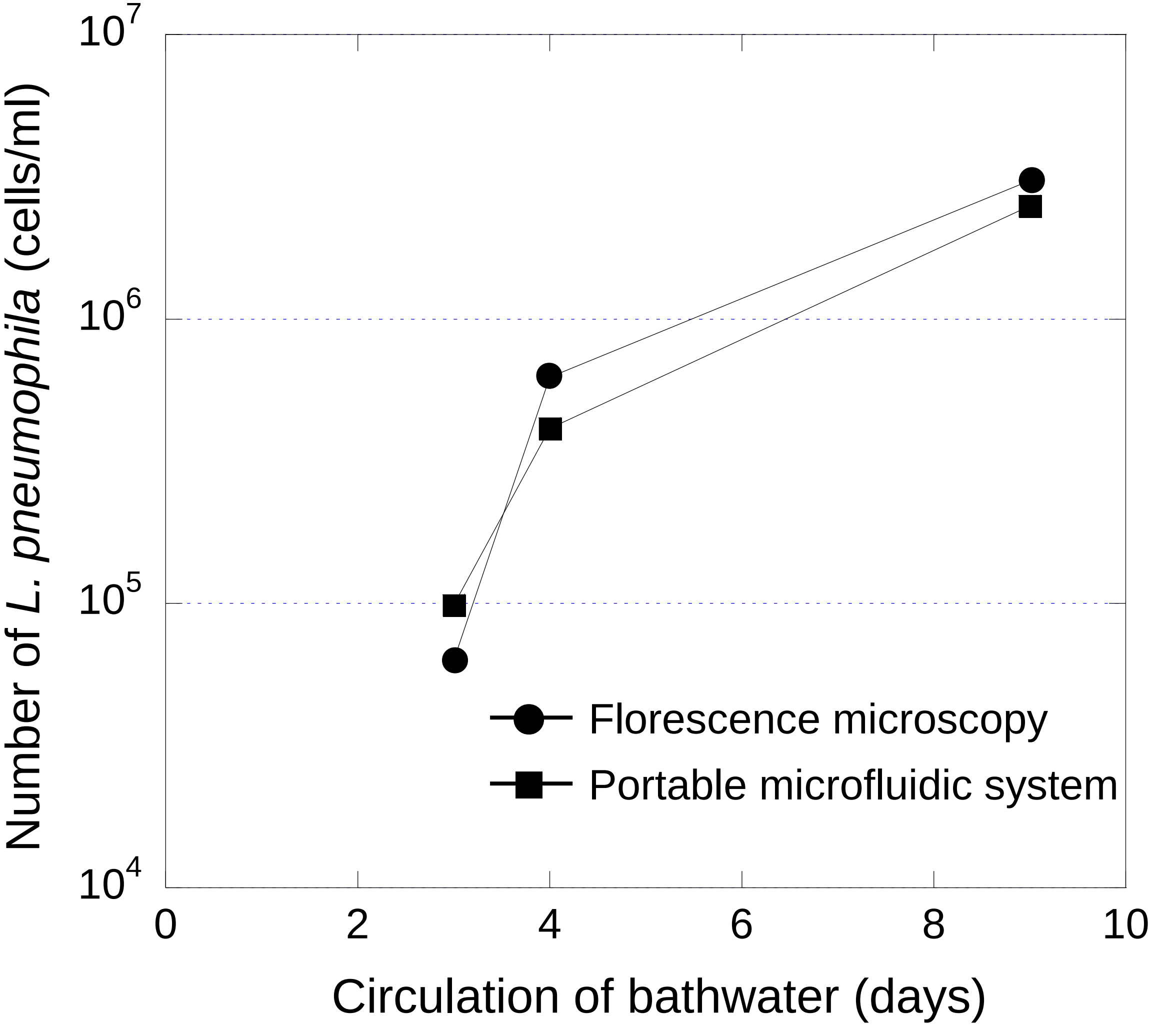


Supplementary Figure S7. Monitoring of *L. pneumophila* in bathtub water by fluorescence microscopy and the portable microfluidic system. A filtration unit equipped in a bath facility, where *L. pneumophila* was detected, were removed and submerged in a test bathtub in the laboratory and then circulation of the water was started. Bathtub water was collected during circulation and the number of *L. pneumophila* cells in the samples were periodically determined by both our microfluidic technique and fluorescence microscopy.

Supplementary Table S1. Microfluidic counts and conventional fluorescence microscopic counts of *L. pneumophila* stained with a fluorescent antibody.

| Fluorescence microscopy | Microfluidic system |
|---|---|
| $(2.3 \pm 1.1^{*}) \times 10^{1}$ | $(1.7 \pm 0.90) \times 10^{1}$ |
| $(1.1 \pm 0.45) \times 10^{2}$ | $(1.3 \pm 0.71) \times 10^{2}$ |
| $(1.1 \pm 0.32) \times 10^{3}$ | $(1.2 \pm 0.47) \times 10^{3}$ |
| $(1.4 \pm 0.62) \times 10^{4}$ | $(1.6 \pm 0.78) \times 10^{4}$ |
| $(1.2 \pm 0.10) \times 10^{5}$ | $(1.4 \pm 0.11) \times 10^{5}$ |
| $(1.1 \pm 0.87) \times 10^{6}$ | $(1.0 \pm 0.92) \times 10^{6}$ |

*: standard deviation
Unit: cells/ml

Cultured *L. pneumophila* cells were spiked in cooling tower water.
Error bars indicate the standard deviation (n=5).
